# Harnessing the multi-targeted potential of rehmanniae radix natural products against renal fibrosis: a mechanistic review

**DOI:** 10.3389/fphar.2025.1690036

**Published:** 2025-11-17

**Authors:** Keqin Zhao, Han Zhu, Liping Zheng, Wenru Wang, Peng Liu, Renhuan Yu, Xiaobei Ma

**Affiliations:** 1 Institute of Basic Theory for Chinese Medicine, China Academy of Chinese Medical Sciences, Beijing, China; 2 Xiyuan Hospital of China Academy of Chinese Medical Sciences, Beijing, China; 3 Department of Postgraduate, Jiangxi University of Chinese Medicine, Nanchang, China; 4 Graduate School, China Academy of Chinese Medical Sciences, Beijing, China

**Keywords:** Rehmanniae Radix, renal fibrosis, CKD, *Verbascoside*, *Catalpol*, *Rehmannioside A*

## Abstract

Renal fibrosis represents the final common pathological pathway of nearly all chronic kidney disease (CKD), yet effective therapeutic options remain profoundly limited. *Rehmanniae Radix*, a botanical drug in Traditional Chinese Medicine (TCM), has a long history of use for its nephroprotective properties. However, a systematic, mechanism-based understanding of how its natural products combat renal fibrosis is conspicuously absent. Herein, we present a comprehensive review to dissect the multi-component, multi-target, and multi-pathway mechanisms through which the major active ingredients of *Rehmanniae Radix* ameliorate renal fibrosis. Our analysis reveals that these natural products, including *Acteoside (also known as Verbascoside)*, *Catalpol*, and *Rehmannioside A*, converge upon the inhibition of the canonical TGF-β1/Smad signaling pathway—a master regulator of fibrosis. This analysis focuses primarily on evidence from preclinical (*in vivo* and *in vitro*) models, as rigorous clinical data on the efficacy of these specific constituents remain limited. Furthermore, they exert potent anti-inflammatory and antioxidant effects via the modulation of pivotal signaling nodes such as NF-κB, Nrf2, and TLR4. Critically, this review illuminates unique and novel mechanisms, including the enhancement of autophagy by *Acteoside* and the targeted inhibition of the AT1R/MAPK14/IL-17 axis by *Rehmannioside A* in hypertensive nephropathy. By elucidating this intricate pharmacological network, this review not only decodes the scientific basis for the nephroprotective effects of *Rehmanniae Radix* but also provides a theoretical foundation for the development of novel anti-fibrotic therapies and identifies promising molecular targets for future investigation.

## 1 Introduction

Chronic kidney disease (CKD) constitutes a formidable global public health challenge. The international consensus paper reports that approximately 700 million people have CKD worldwide (∼9% of the global population) (Coresh et al., 2007; Webster et al., 2017; Chen et al., 2024; Francis et al., 2024). Prevalent etiologies such as diabetes, hypertension, and various forms of nephritis are major drivers of renal fibrosis. This pathology is characterized by a loss of the peritubular capillary network, a progressive accumulation of extracellular matrix (ECM) proteins, and the infiltration of activated myofibroblasts and inflammatory cells. These structural changes precipitate a relentless decline in renal function, marked by diminished renal blood flow (RBF) and tissue perfusion, a reduced glomerular filtration rate (GFR), impaired tubular handling of water and electrolytes, and elevated proteinuria (Ma et al., 2023; Han et al., 2025; jia et al., 2024). Regardless of the initial etiology, the final common pathway for most progressive CKD is renal fibrosis, a pathological process for which therapeutic interventions remain remarkably scarce (Patera et al., 2024).

The progression of CKD is underpinned by a complex interplay of mechanisms. At the phenotypic level, these include chronic inflammation, driven by factors such as M1 macrophage polarization or complement system dysregulation (Hu et al., 2024); excessive ECM deposition, the hallmark of fibrosis (Panizo et al., 2021); cellular energy metabolism collapses due to oxidative stress and mitochondrial dysfunction (Wang H. et al., 2024; Młynarska et al., 2024); metabolic reprogramming, including aberrant renal lipid metabolism (Wang Y. et al., 2024); and dysbiosis of the gut microbiota (Wang H. et al., 2023). Deeper investigation has revealed that epigenetic modifications also play a critical role. For instance, promoter regions of anti-fibrotic genes like *KLOTHO* can be silenced via hypermethylation, while pro-fibrotic genes may be activated through hypomethylation, thereby promoting fibrosis (Zhang Y. et al., 2024; Jin et al., 2024). Moreover, histone deacetylases (HDACs) can facilitate fibrotic progression by deacetylating anti-fibrotic gene histones, further fueling CKD advancement (Jin et al., 2024).

As the culminating pathological stage of CKD, fibrosis is orchestrated by several key signaling cascades, most notably the TGF-β/Smad, Wnt/β-catenin, Notch, and Renin-angiotensin-aldosterone system (RAAS) pathways (Liu, 2024; Liu et al., 2022). While modern medicine has made strides in managing the primary diseases leading to CKD, specific therapeutic interventions targeting the core fibrotic process remain remarkably scarce.

The Dapagliflozin and Prevention of Adverse Outcomes in Chronic Kidney Disease (DAPA-CKD trial) demonstrated that dapagliflozin significantly reduced the composite endpoint of “≥50% eGFR decline, End-stage kidney disease (ESKD), or renal/cardiovascular death” (HR ≈ 0.61) in both diabetic and non-diabetic CKD populations, and slowed the change in eGFR slope (Heerspink et al., 2020). The Study of Heart and Kidney Protection With Empagliflozin (EMPA-KIDNEY trial) confirmed that empagliflozin reduced the risk of kidney disease progression or cardiovascular death in a broader range of CKD patients (The EMPA-KIDNEY Collaborative Group et al., 2023). Mechanistically, Sodium-glucose cotransporter 2 (SGLT2) inhibitors restore tubuloglomerular feedback, reduce intraglomerular pressure, improve proximal tubular metabolic stress, and are associated with indirect inhibition of pro-inflammatory/pro-fibrotic signaling (such as alleviating interstitial inflammation and oxidative stress), thereby slowing structural progression (Stevens et al., 2024; Huang et al., 2023). Regarding applicability, SGLT2 inhibitors can be added to Angiotensin-converting enzyme inhibitor (ACEi)/Angiotensin II receptor blocker (ARB) therapy; however, the “physiological” decline in eGFR early in treatment, genitourinary infections, volume depletion, and rare ketoacidosis require timely recognition and intervention by clinicians (Heerspink et al., 2020; The EMPA-KIDNEY Collaborative Group et al., 2023; Stevens et al., 2024). The Finerenone in reducing Kidney Failure and Disease Progression in Diabetic Kidney Disease (FIDELIO-DKD) and Finerenone in Reducing Cardiovascular Mortality and Morbidity in Diabetic Kidney Disease (FIGARO-DKD) trials demonstrated the beneficial effects of finerenone on renal/cardiovascular outcomes in populations with more severe and mild-to-moderate DKD, respectively; when combined with standard RAAS inhibitors, finerenone further reduced residual risk (Bakris et al., 2020; Van Mieghem et al., 2021). The individual patient-level pooled analysis from Finerenone in chronic kidney disease and type 2 diabetes: Combined FIDELIO-DKD and FIGARO-DKD Trial programme analysis (FIDELITY) reinforced the consistent benefits of finerenone on both renal and cardiovascular outcomes (Agarwal et al., 2022). At the biomarker level, reductions in Urine albumin-to-creatinine ratio (UACR) and improvement in eGFR slope support the anti-inflammatory/anti-fibrotic effects of finerenone; however, the increased risk of hyperkalemia requires careful consideration, especially in patients with low eGFR, high UACR, and concomitant RAAS inhibition (Stevens et al., 2024; Bakris et al., 2020; Van Mieghem et al., 2021; Agarwal et al., 2022). RAAS inhibition remains a cornerstone therapy, but evidence for “fibrosis reversal” is limited, and its anti-fibrotic efficacy needs improvement (Stevens et al., 2024). In the Evaluate Renal Function With Semaglutide Once Weekly (FLOW) trial, Glucagon-like peptide-1 receptor agonists (GLP-1Ras) reduced the risk of major kidney disease events by approximately 20%–24% and slowed the annual rate of eGFR decline, suggesting potential value in targeting the metabolic-inflammatory axis for anti-renal fibrosis. Current analyses are uncertain about the effects of combination therapy with SGLT2 inhibitors, requiring more stratified and synergistic studies (Perkovic et al., 2024; Mann et al., 2024). Novel molecules targeting core fibrotic pathways (such as TGF-β/SMAD, epigenetic modifications, and immune-metabolic coupling) are still in early/translational stages, lacking validation with hard endpoints and histology (Huang et al., 2023; Li et al., 2022). Current standard-of-care drugs, such as angiotensin-converting enzyme inhibitors (ACEIs), can slow the deterioration of renal function in some patients, but they fail to halt or reverse the fibrotic cascade entirely, with a large proportion of patients ultimately succumbing to ESKD.

This pressing clinical need has catalyzed the search for more precise and potent anti-fibrotic agents. The TGF-β/Smad pathway has long been recognized as the ‘master regulator’ driving renal fibrosis, making it a prime target for drug development, exemplified by the Smad3 inhibitor Specific Inhibitor of Smad3 (SIS3) (Zhang et al., 2018). However, given the pleiotropic roles of Transforming growth factor-beta (TGF-β) in physiological homeostasis, complete blockade of this pathway carries the risk of significant adverse effects (Hoelder et al., 2012). Consequently, research has pivoted towards targeting downstream or parallel effectors. Promising examples include VCP979, a p38 MAPKα inhibitor that mitigates renal fibrosis in unilateral ureteral obstruction (UUO) rats through anti-inflammatory and anti-fibrotic actions (Min et al., 2023), and INS018_055, a Traf2- and Nck-interacting kinase (TNIK) inhibitor that has demonstrated broad-spectrum anti-fibrotic activity in preclinical models of kidney, lung, and skin fibrosis (Ren et al., 2025). The multi-target tyrosine kinase inhibitor nintedanib, approved for idiopathic pulmonary fibrosis (IPF), has also shown efficacy in suppressing renal fibrosis, while imatinib is in Phase III trials for nephrogenic systemic fibrosis (Qin et al., 2022; Pu et al., 2013). The Akt/mTOR pathway, a central hub for cell growth and survival, can be targeted by inhibitors like MK-2206, which has ameliorated fibrosis in multiple animal models of kidney injury (Chen et al., 2022). Furthermore, inhibitors of the Bromodomain and extraterminal domain (BET) protein family, such as I-BET151 and JQ1, have shown significant anti-fibrotic potential by disrupting the “reading” of acetylated histones that drives pro-inflammatory and pro-fibrotic gene expression (Zhou et al., 2017; Xiong et al., 2016). The Inducible nitric oxide synthase (iNOS) inhibitor SKLB023 suppresses the expression of Alpha-smooth muscle actin (α-SMA), collagens, and fibronectin and reduces TGF-β1/Smad3 phosphorylation in the UUO model, highlighting its therapeutic potential (Feng et al., 2018). Targeting the immune-fibrosis crosstalk, the Signal transducer and activator of transcription 6 (STAT6) inhibitor AS1517499 has been shown to improve renal fibrosis by inhibiting myeloid fibroblast activation (Jiao et al., 2021). Despite this wealth of promising preclinical data, a formidable hurdle remains: translating the remarkable efficacy observed in animal models into tangible clinical benefits for patients. Many novel targets are fundamental to cellular life, and the potential for off-target effects and systemic side effects from long-term inhibition remains a primary obstacle in drug development.

In this context, TCM has emerged as a rich reservoir of potential therapeutic strategies, with many herbal formulas shown to attenuate renal fibrosis by exerting anti-inflammatory, antioxidant, and fibroblast-inhibiting effects (Liu et al., 2021; Xu et al., 2025). It should be noted that the concept of “kidney” in TCM does not refer solely to the “kidney organ,” but rather encompasses a wide range of physiological functions in the human body, including but not limited to growth, development, reproduction, and fluid metabolism. The concept of “kidney fibrosis” cannot be closely correlated with the TCM concept of “kidney.” However, based on the therapeutic characteristics of “concept of holism” and “syndrome differentiation and treatment”. TCM can utilize traditional Chinese medicinal botanical drugs to exert anti-fibrotic effects on the kidneys. The ancient TCM classic “Sheng nong’s herbal classic” records that “It tastes sweet and is cold, non-toxic. It is used for treating fractures, muscle injuries, blood stasis, replenishing bone marrow, and promoting muscle growth. When decocted, it can eliminate cold and heat, accumulations, and arthralgia. The fresh one is particularly effective. Long-term use can lead to a light body and anti-aging effects.” Since ancient times, TCM has used Rehmannia as a common medicine for strengthening the kidneys and treating kidney diseases. With the development of modern science and technology, the mechanisms of action of Rehmannia and traditional Chinese medicine formulas containing Rehmannia in treating kidney diseases are gradually being revealed.

Among these, *Rehmanniae Radix* (*Sheng Dihuang*) is a widely utilized component in anti-fibrotic TCM prescriptions. First, documented in the *Shen Nong Ben Cao Jing* (*The Divine Farmer’s Materia Medica*), *Rehmanniae Radix* is defined by the Chinese Pharmacopoeia as the fresh or dried root tuber of *Rehmannia glutinosa Libosch*, prepared by a slow baking process. For instance, the polyherbal formula ‘Qidan Dihuang Decoction’ (*Qidan Dihuang Tang*) effectively alleviates diabetic kidney injury and fibrosis by inhibiting the PERK-eIF2α-ATF4 pathway and promoting autophagy (Liang et al., 2022). Similarly, Renshen Guben oral liquid (RSGB), a formula approved by the National Medical Products Administration (NMPA) of China, has been shown to mitigate renal fibrosis via the TGFβ1/Smad2/3, Wnt4/β-catenin, and NGFR/NF-κB pathways (Zhang et al., 2023). Notably, *Rehmanniae Radix* is a principal component in these and many other nephroprotective formulations, suggesting its pivotal role in their therapeutic effects.

Currently, research on the anti-fibrotic effects of *Rehmanniae Radix* has predominantly focused on its use within complex formulas, leaving a conspicuous gap in our understanding of the specific contributions and mechanisms of its individual natural active products. Given the pivotal role of Rehmanniae Radix in numerous nephroprotective formulations, a systematic, mechanism-based analysis of its individual bioactive constituents is urgently needed. This review aims to fill this gap. We will first outline the key pathophysiological mechanisms driving renal fibrosis. Then, we will systematically dissect how the major natural products from Rehmanniae Radix, such as Catalpol, Acteoside, and Rehmannioside A *(ReA)*, modulate these pathways. Finally, we will provide a critical appraisal of the current evidence, identify limitations, and propose future research priorities. The literature search strategy employed for this review is detailed in the following section(summarized in Table 1).

**TABLE 1:** Research progress on the anti-renal fibrosis effects of natural products in *Rehmannia glutinosa*.

Active ingredient	Experimental model	Test concentration/Dose	Pharmacological mechanism	Specific molecular target	Reference
Acteoside	*In vivo: Adenine-induced CKD rats;* *In vitro: IAA-induced NRK-52E cells*	*In vivo*: 40 mg/kg/day; *In vitro*: 10–80 μΜ	Anti-inflammatory/antioxidant	AHR(↓), NF-κB p65(↓), Nrf2(↑)	Wang et al. (2025)
	*In vivo: UUO rats model*	40 mg/kg/day	Anti-inflammatory/antioxidant	HMGN1(↓), TLR4(↓), TREM-1(↓), F4/80(↓), α-SMA(↓)	Mao et al. (2023)
	*In vivo: Unilateral nephrectomy + STZ-induced rats;* *In vitro: High glucose + H2O2-treated HK-2 cells*	*In vivo*: 50 mg/kg/day; *In vitro*: 0.25–1.0 μΜ	Promote autophagy/antioxidant	TFEB(↑), ROS(↓), LC3(↑), p62(↓)	Zhou et al. (2024)
Catalpol	*In vivo: AA-I induced C57BL/6N mice;* *In vitro: AA-I induced NRK-52E cells*	*In vivo*: 10, 100 mg/kg; *In vitro*: 5, 10 μΜ	Anti-inflammatory/antioxidant	Nrf2(↑), NF-κB p65(↓), IL-6(↓), Cleaved-Caspase3(↓)	Liu et al. (2024)
	*In vivo: Ang II induced C57BL/6J mice;* *In vitro: Ang II treated SV40 MES 13, NRK-49F, HK-2 cells*	*In vivo*: 25–100 mg/kg; *In vitro*: 1–10 μΜ	Anti-inflammatory/antifibrotic	p-NF-κB p65(↓), TGF-β1(↓), p-Smad2/3(↓)	Cong et al. (2022)
	*In vivo: Adenine-induced BALB/c mice*	5 mg/kg (5 days/week)	Anti-inflammatory (Sirt1 activation)	Sirt1(↑), p-NF-κB p65(↓), TNF-α(↓), IL-6(↓)	Zaaba et al. (2023)
Rehmannioside A	*In vivo: Ang II induced C57BL/6J mice;* *In vitro: Ang II treated NRK-52E cells*	*In vivo*: 30–120 mg/kg/d; *In vitro*: 20–80 μΜ	Anti-inflammatory/Regulate the renin-angiotensin-aldosterone system (RAAS)	AT1R(↓), MAPK14(↓), IL-17(↓), p-NF-κB p65(↓)	Liu et al. (2025)
Echinacoside	*In vivo: db/db mice*	300 mg/kg/day	Antifibrotic	TGF-β1(↓), Smad2/3/4(↓), FN(↓), Collagen IV(↓), α-SMA(↓)	Tang et al. (2017)
γ-Aminobutyric Acid	*In vivo: Optimized 5/6 nephrectomy mouse model*	100, 500 mg/kg	Antifibrotic	TGF-β1(↓), Fibronectin(↓)	Sasaki et al. (2007)
Salidroside/Rhodioloside	*In vivo: UUO/FA induced mice;* *In vitro: TGF-β1 induced HK-2 cells*	*In vivo*: 40, 80 mg/kg; *In vitro*: 2–50 μΜ	Anti-inflammatory	TLR4(↓), p-NF-κB(↓), JNK(↓), Erk(↓), P38(↓)	Li et al. (2019)
	*In vivo: STZ-induced rats*	100 mg/kg	Antifibrotic	Wnt1(↓), Wnt3a(↓), TGF-β1(↓), Smad-3(↓), β-catenin(↓)	Shati and Alfaifi (2020)
	*In vivo: HFD/STZ-induced rats*	50, 100 mg/kg/day	Metabolic regulation (Sirt1 activation)	Sirt1(↑), PGC-1α(↑)	Xue et al. (2019)

(“↑, increased; ↓, decreased”).

## 2 Literature search and selection strategy

A comprehensive literature search was conducted across multiple electronic databases, including PubMed, Scopus, and Web of Science, to identify relevant studies published up to July 2025. The search strategy employed a combination of keywords and MeSH terms, such as (“rehmannia root” [Supplementary Concept]) OR ((((((rehmanniae radix[Title/Abstract]) OR (sheng-di-huang[Title/Abstract])) OR (radix rehmanniae[Title/Abstract])) OR (radix rehmanniae preparate[Title/Abstract])) OR (Rehmannia decoction[Title/Abstract])) OR (rehmannia root preparation[Title/Abstract])), in combination with the names of its primary bioactive constituents: “Catalpol”, “Acteoside” OR “Verbascoside”, and “Rehmannioside A”. The search was limited to original research articles published in English that investigated the molecular mechanisms of these compounds in preclinical (*in vivo* animal and *in vitro* cellular) models of renal fibrosis. Studies were included only if they provided clear, mechanism-based data. Narrative reviews, conference abstracts, non-English articles, and studies lacking mechanistic investigation were excluded from this review. This review was prepared adhering to the principles outlined in the Four Pillars of Best Practice for Ethnopharmacology Research (Heinrich et al., 2022).

## 3 The pathophysiological landscape of renal fibrosis

### 3.1 The TGF-β1/Smad pathway: the master regulator

The TGF-β1/Smad signaling cascade is the canonical axis driving the pathogenesis of renal fibrosis. As a master regulator, TGF-β1 initiates the myofibroblastic transdifferentiation of renal parenchymal cells, fueling the excessive deposition of ECM components like collagens and fibronectin (Zhang et al., 2021; Liu et al., 2023). This process is transduced through the phosphorylation of Smad2/3, which forms a complex with Smad4 to regulate the transcription of pro-fibrotic genes (Lai et al., 2024). Consequently, blockade of TGF-β signaling has emerged as a promising therapeutic strategy. Fresolimumab, a humanized monoclonal antibody against TGF-β, has been shown to reduce TGF-β expression and type III collagen deposition, ameliorate tubular apoptosis, and restore tissue oxygenation in a mouse model of cyclosporine nephropathy (Ling et al., 2003). The clinical relevance of this pathway is underscored by therapeutic agents like fresolimumab, a TGF-β-neutralizing antibody that has shown potential in mitigating the eGFR decline in patients with primary Focal segmental glomerulosclerosis (FSGS) (Trachtman et al., 2011; Vincenti et al., 2017). While this validates the pathway as a drug target, it also highlights the need for novel modulators.

### 3.2 Sterile inflammation and immune crosstalk

Emerging evidence highlights the HMGN1/TLR4/TREM-1 pathway as a critical axis linking tissue injury signals to the downstream cascade of sterile inflammation and fibrosis in the kidney. In non-infectious renal injuries stemming from metabolic disorders, ischemia-hypoxia, or toxins, this “sterile inflammation,” driven by endogenous molecules, plays a paramount role (Yu et al., 2024). During the pathogenesis of diabetic kidney disease (DKD), Damage-Associated Molecular Patterns (DAMPs) released from stressed or dying cells initiate a pro-inflammatory cascade (Wang Y. et al., 2023; Tang and Yiu, 2020). These endogenous molecules trigger the nuclear translocation of transcription factors like NF-κB, promoting the production of pro-inflammatory cytokines (Wada and Makino, 2016; Gong et al., 2020).

A key pathogenic event in renal fibrosis is the inflammatory crosstalk between damaged parenchymal cells and innate immune sentinels, a dialogue mediated by the HMGN1/TLR4/TREM-1 axis. Under sterile injury conditions, nuclear HMGN1 is released as an alarmin by stressed tubular cells, where it subsequently engages TLR4 on resident and infiltrating myeloid cells (Alam et al., 2018; Yang et al., 2012; Yu et al., 2018; Yu et al., 2019). This signal is then amplified via TREM-1, a receptor predominantly expressed on myeloid cells, creating a potent inflammatory feed-forward loop that drives tissue destruction and fibrosis (Lo et al., 2014). This axis therefore represents a critical interface between tissue damage and the innate immune response.

Following kidney injury, the initiation of the fibrotic process begins with the precise recruitment of immune cells to the site of damage, where the signaling axis formed by C-C motif chemokine ligand 2 (CCL2) and its receptor CCR2 plays a critical “call to arms” role (Guo et al., 2024). Particularly in DKD, tubular epithelial cells significantly upregulate the expression and secretion of CCL2 after injury (Bhatia et al., 2019). Elevated CCL2 exacerbates macrophage infiltration in the glomerular and tubulointerstitial regions (Sullivan et al., 2013), and recruits CCR2-positive monocytes to the injured kidney, promoting their differentiation into pro-inflammatory M1 macrophages (Bhatia et al., 2019). The polarization of M1 macrophages is primarily driven by signals such as tumor necrosis factor-α (TNF-α) and interferon-γ (IFN-γ) (Ma et al., 2025). Concurrently, M1 macrophages produce and release large amounts of pro-inflammatory cytokines, including TNF-α, interleukin-1α (IL-1α), IL-1β, and IL-6, while also generating reactive oxygen species (ROS) and activating the NLRP3 inflammasome, further amplifying inflammatory signals and promoting subsequent fibrotic progression (Zeng et al., 2024). As the disease progresses, changes in the renal microenvironment promote the transition of macrophages from the pro-inflammatory M1 phenotype to the anti-inflammatory M2 phenotype. Although M2 macrophages play a role in tissue healing and inflammation resolution during the repair phase of acute injury, they are also a major source of TGF-β, which is currently recognized as the most potent pro-fibrotic cytokine (Zeng et al., 2024). In the context of chronic inflammation, large amounts of TGF-β secreted by M2 macrophages greatly promote ECM synthesis and deposition by activating resident fibroblasts and pericytes, and potentially inducing the transdifferentiation of tubular epithelial cells and macrophages themselves into α-SMA-expressing myofibroblasts (Wang et al., 2021). Myofibroblasts are the main producers of fibrotic scar tissue, and their sustained activation is key to the irreversibility of renal fibrosis.

In various kidney diseases, Th17 cells are found to be abnormally active, becoming a key force driving inflammation and fibrosis (Basile et al., 2021). The primary member of the IL-17 family is IL-17A, whose receptors are widely expressed on various cell types in the kidney, including podocytes and tubular epithelial cells, making it a broadly acting effector molecule (Paquissi and Abensur, 2021). Compared to macrophages, T helper cells amplify and sustain the pro-fibrotic inflammatory environment through more specific and persistent responses. In various kidney diseases, Th17 cells are found to be abnormally active, becoming a key force driving inflammation and fibrosis (Basile et al., 2021). The primary member of the IL-17 family is IL-17A, whose receptors are widely expressed on various cell types in the kidney, including podocytes and tubular epithelial cells, making it a broadly acting effector molecule (Paquissi and Abensur, 2021). The Th17/IL-17 axis promotes renal fibrosis through multiple mechanisms. Firstly, IL-17A can directly act on tubular epithelial cells, activating pro-fibrotic pathways, such as upregulating TGF-β expression, and promoting Epithelial-Mesenchymal Transition (EMT), a process where epithelial cells acquire mesenchymal characteristics and begin producing ECM 21. Secondly, IL-17A is a potent amplifier of pro-inflammatory signals. It stimulates resident kidney cells and other immune cells to synthesize and release more pro-inflammatory cytokines and chemokines, which not only exacerbates local inflammation but also leads to granulopoiesis/myelopoiesis, thereby recruiting more neutrophils and monocytes into the kidney, forming a vicious cycle (Basile et al., 2021).

### 3.3 Oxidative stress and the Nrf2 antioxidant system

Nuclear factor erythroid 2-related factor 2 (Nrf2) is a master transcription factor that orchestrates the cellular defense against oxidative damage by regulating the expression of a suite of antioxidant genes (Zheng et al., 2024). The critical role of Nrf2 in renal protection is well-established. For instance, a study by Qiao et al. (2023) demonstrated that Nrf2 knockout exacerbated renal injury in a mouse model of hyperuricemic nephropathy (HN), whereas pharmacological activation of Nrf2 ameliorated renal dysfunction and fibrosis. Mechanistically, Nrf2 activation mitigated oxidative stress by restoring mitochondrial homeostasis, reducing NADPH oxidase 4 (NOX4) expression, and upregulating downstream targets like heme oxygenase-1 (HO-1) and quinone oxidoreductase 1 (NQO1). Furthermore, active Nrf2 signaling attenuated renal fibrosis by downregulating the TGF-β1 pathway. Similarly, in the context of Lupus Nephritis (LN), elevated renal Nrf2 expression in patients suggests an active but potentially insufficient antioxidant response to glomerular oxidative stress. In a corresponding pristane-induced murine model, Nrf2-deficient mice exhibited hyperactivation of NF-κB and TGF-β1 signaling, leading to increased expression of TGF-β1, fibronectin, and iNOS, and culminating in more severe renal injury and fibrosis compared to their wild-type counterparts (Jiang et al., 2014).

### 3.4 Impaired autophagy and cellular homeostasis

Dysfunctional autophagy is increasingly recognized as a central driver of the sterile inflammation that underpins renal fibrosis. This process is governed by the master transcriptional regulator Transcription factor EB (TFEB), which orchestrates the expression of genes essential for both autophagy and lysosomal biogenesis (Li and Wang, 2023). In fibrotic kidneys, TFEB activity is pathologically suppressed by hyperactive Mammalian target of rapamycin complex 1 (mTORC1) signaling, leading to an accumulation of damaged organelles and protein aggregates that can act as endogenous damage-associated molecular patterns (DAMPs) (Ren et al., 2024). The therapeutic potential of reversing this state is profound; restoring TFEB function via genetic tools has been shown to halt fibrotic progression, validating it as a high-value target.

### 3.5 Role of other key signaling pathways

The Renin-Angiotensin System (RAS) plays a critical role in the pathogenesis of hypertensive nephropathy, with the primary effector peptide Angiotensin II (Ang II) being particularly important (Ruiz-Ortega et al., 2006). Ang II, by activating the AT1 receptor (AT1R), triggers inflammatory cell infiltration in renal tissue, upregulates pro-inflammatory factors, and exacerbates oxidative stress responses (Ruiz-Ortega et al., 2006; Nguyen et al., 2013). Ang II can also induce the overexpression of pro-fibrotic factors TGF-β1 and Connective Tissue Growth Factor (CTGF) (Wolf, 2006; Rupérez et al., 2003), where TGF-β1 drives ECM deposition via the Smad pathway, and CTGF, as its downstream molecule, synergistically enhances the production of ECM components such as Collagen Type I and fibronectin (Wolf, 2006; Rupérez et al., 2003). Therefore, Ang II-mediated inflammation, oxidative stress, and factors like TGF-β1/CTGF collectively promote the progression of hypertension-related renal fibrosis (Ruiz-Ortega et al., 2006; Wolf, 2006; Rupérez et al., 2003).

The Wnt/β-catenin signaling pathway exhibits a “double-edged sword” effect in kidney development and injury repair: while crucial for normal organogenesis during embryonic development, its activity is minimal in the mature kidney; however, upon injury, it is rapidly and aberrantly activated, inducing the transdifferentiation of renal interstitial fibroblasts into myofibroblasts, which possess enhanced migratory and proliferative capabilities and synthesize large amounts of ECM. Simultaneously, Wnt/β-catenin signaling can directly upregulate the expression of ECM genes like Collagen Type I and fibronectin, leading to progressive ECM deposition and scar formation in renal tissue. Studies in animal models have shown that blocking the Wnt/β-catenin pathway (e.g., using its antagonist Dickkopf-1) can inhibit myofibroblast activation and reduce collagen and fibronectin deposition, thereby alleviating the degree of renal fibrosis (Tan et al., 2014).

### 3.6 Key experimental models for studying renal fibrosis

In order to study the complex mechanisms of renal fibrosis and to assess the efficacy of potential therapies, researchers have developed a variety of preclinical experimental models. The following is a brief introduction to several major models covered in this review.

UUO is a model induced by surgically ligating one ureter, characterized by the rapid and reproducible induction of significant tubulointerstitial fibrosis and inflammation; its advantages include rapidity and stability, but a limitation is that its etiology (complete obstruction) differs from common causes of human CKD (such as diabetes and hypertension) (Chevalier et al., 2009; Na et al., 2004). Second is the 5/6 Nephrectomy (5/6 Nx) model, which reduces nephron number by removing one kidney and a large portion of the contralateral kidney; it simulates the progressive loss of renal function accompanied by hypertension, proteinuria, and slowly developing fibrosis. Its advantage lies in better mimicking the progressive nature of human CKD, while drawbacks include greater model variability and slower fibrosis development (Bener et al., 2012). Next are Diabetic Nephropathy Models, commonly established using methods like streptozotocin (STZ)-induced type 1 diabetes models or genetic models of diabetes (such as db/db mice); these models simulate the hyperglycemia, glomerular lesions, proteinuria, and eventual fibrosis caused by diabetes. Their strength is the etiological relevance to the most common cause of human CKD, but limitations include model complexity and potentially late or non-significant fibrosis development (Tesch and Allen, 2007; Sharma et al., 2003). Lastly, Nephrotoxic Models include, for example, the adenine-induced model (which causes chronic injury via crystal formation obstructing renal tubules) or drug-induced models (such as with cyclosporine A or cisplatin); these simulate kidney injury and fibrosis caused by specific toxins or drugs. Their advantage is the ability to study specific injury mechanisms, while a limitation is that the pattern of injury may differ from common CKD etiologies (Jia et al., 2013).

## 4 Multi-targeted regulation of renal fibrosis by natural products from rehmanniae radix

### 4.1 Modulation of the TGF-β1/Smad pathway

**FIGURE 1:**
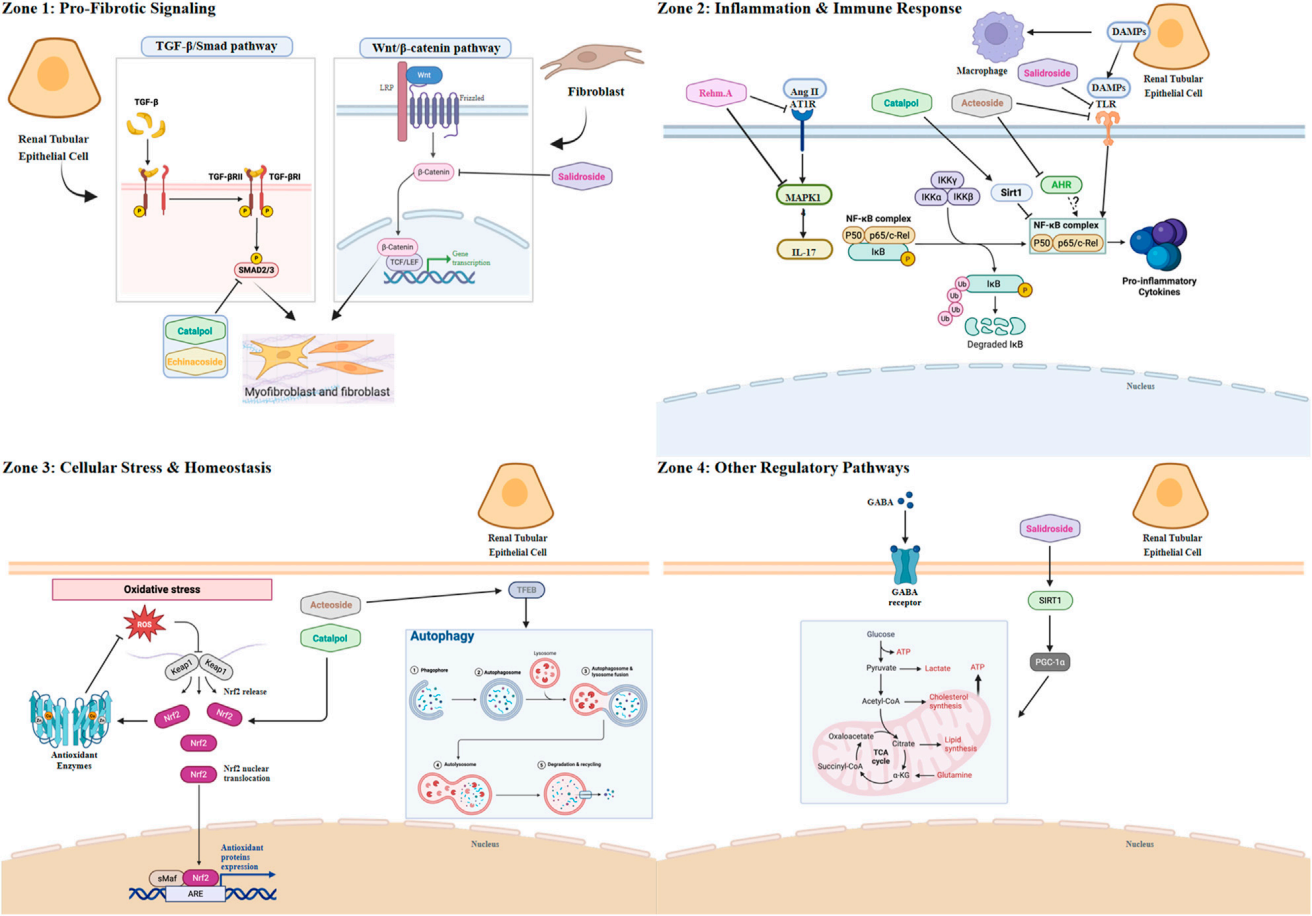
The multi-target mechanistic network of key natural products from *Rehmanniae Radix* against renal fibrosis. Schematic overview illustrating the complex interplay between renal parenchymal cells (e.g., Tubular Epithelial Cells, TECs), stromal cells (e.g., Fibroblasts/Myofibroblasts), and immune cells (e.g., Macrophages) during the progression of renal fibrosis. The diagram highlights key pathological signaling pathways organized by major mechanistic categories and depicts the specific molecular targets modulated by major natural products derived from *Rehmanniae Radix*. Zone 1: Pro-fibrotic Signaling. This zone depicts pathways primarily active in fibroblasts/myofibroblasts and TECs that directly drive extracellular matrix (ECM) deposition. Transforming growth factor-beta 1 (TGF-β1) activates its receptors (TGF-β-R) leading to phosphorylation of Smad2/3 (p-Smad2/3), a key step in fibrosis induction. Both Catalpol and Echinacoside inhibit this pathway, targeting the phosphorylation of Smad2/3. Additionally, aberrant activation of the Wnt/β-catenin pathway in fibroblasts contributes to ECM production. Salidroside has been shown to suppress Wnt/β-catenin signaling. Zone 2: Inflammation and Immune Response. This zone focuses on pathways driving sterile inflammation, primarily involving macrophages but also TECs and T cells. The central node NF-κB integrates multiple upstream signals leading to pro-inflammatory cytokine production. Sirtuin 1 (Sirt1) normally suppresses NF-κB; Catalpol activates Sirt1. The aryl hydrocarbon receptor (AHR) also modulates NF-κB; Acteoside inhibits AHR. Damage-associated molecular patterns (DAMPs) like HMGN1 activate Toll-like receptor 4 (TLR4) and TREM-1 on macrophages, activating NF-κB; this axis is inhibited by Acteoside and Salidroside. The Renin-Angiotensin System (RAS) effector Angiotensin II (Ang II) acts via the AT1 receptor (AT1R) and MAPK14 to induce IL-17 (potentially from T cells), further activating NF-κB; Rehmannioside A inhibits both AT1R and MAPK14. Zone 3: Cellular Stress and Homeostasis. This zone illustrates pathways within TECs managing cellular stress and maintaining balance. Oxidative stress (represented by ROS) is counteracted by the Nrf2 pathway, which induces antioxidant enzymes (e.g., HO-1, NQO1). Catalpol activates Nrf2. Cellular damage is cleared via autophagy, a process regulated by TFEB. Acteoside promotes autophagy by activating TFEB. Zone 4: Other Regulatory Pathways. This zone depicts additional mechanisms primarily in TECs. The inhibitory neurotransmitter GABA acts via GABA receptors (GABA-R); GABA (as a compound) modulates this system. Mitochondrial biogenesis and health, crucial for cell resilience, are promoted by the Sirt1/PGC-1α axis; Salidroside activates this pathway. Created in BioRender. Zhao, K. (2025) https://BioRender.com/3n7k0jp

*Catalpol*, an iridoid glycoside, stands as a primary natural products of *Rehmannia glutinosa*. Its pharmacological profile is well-documented, encompassing potent anti-inflammatory, antioxidant, anti-apoptotic, and neurotrophic properties (Bai et al., 2019; Yang et al., 2020). In the context of metabolic disease, *Catalpol* exerts hypoglycemic effects, attributed to its capacity to restore mitochondrial function and enhance glucose uptake via increased Glucose transporter type 4 (GLUT4) expression (Li et al., 2014; Bhattamisra et al., 2020). These foundational activities translate into significant nephroprotection, particularly in diabetic nephropathy, where it has been shown to mitigate renal injury by reducing serum creatinine (Scr) and proteinuria, alleviating oxidative stress, and attenuating podocyte apoptosis (Fu et al., 2023). *Catalpol’s* anti-fibrotic effects on the kidney are shown in Figure 1.

The role of Catalpol in this pathway was rigorously investigated by Cong et al. in both Ang II-infused mice and *in vitro* cell models (SV40 MES 13, NRK-49F, and HK-2) (Cong et al., 2022). The study provided direct phenotypic evidence of nephroprotection: Catalpol treatment was shown to significantly attenuate the expression of key fibrotic markers, specifically Collagen IV and fibronectin. Mechanistically, this anti-fibrotic effect was subsequently linked to the inhibition of the canonical pathway, as evidenced by the suppression of both TGF-β1 expression and the downstream phosphorylation of Smad2/3. These findings suggest that *Catalpol* interferes with the core machinery of the TGF-β1/Smad pathway, positioning it as a compelling therapeutic candidate for further mechanistic dissection.

The role of Echinacoside, another phenylethanoid glycoside analogue of *Acteoside*, in this pathway was rigorously investigated by Tang et al. in diabetic db/db mice and by You et al. in TGF-β1–stimulated HSC-T6 cells (You et al., 2016; Tang et al., 2017). The studies provided direct phenotypic evidence of nephroprotection: in db/db mice, Echinacoside significantly reduced interstitial fibrosis and matrix accumulation and attenuated the expression of key fibrotic markers—Collagen IV and fibronectin—with concurrent improvements in albuminuria and renal function indices. Mechanistically, this anti-fibrotic effect was linked to inhibition of the canonical pathway, as evidenced *in vivo* by suppression of TGF-β1 and Smad2/3 and *in vitro* by reduced p-Smad2/3 with upregulated Smad7. Collectively, these findings indicate that Echinacoside interferes with the core machinery of the TGF-β1/Smad axis, supporting its candidacy for further mechanistic dissection.

*Echinacoside*, a phenylethanoid glycoside analogue of *Acteoside*, provides mechanistic reinforcement by also targeting the canonical TGF-β1/Smad signaling cascade. Its demonstrated ability to preserve renal architecture and podocyte integrity in diabetic nephropathy underscores the herb’s layered strategy for suppressing this central pro-fibrotic axis.

### 4.2 Attenuation of sterile inflammation and immune responses

A study by Zaaba et al. (2023) provided compelling evidence that *Catalpol* engages this protective axis. In a mouse model of adenine-induced chronic kidney disease, *Catalpol* administration robustly attenuated inflammation, oxidative stress, and fibrosis. Mechanistically, these effects were directly linked to the activation of Sirt1 and the consequent inhibition of downstream NF-κB signaling. This pivotal finding, however, opens a critical line of inquiry: does *Catalpol* act as a direct allosteric activator of Sirt1, or does it operate upstream, perhaps by augmenting the cellular NAD^+^ pool, thereby enhancing Sirt1’s catalytic activity? Elucidating this precise molecular interaction is the essential next step in validating *Catalpol* as a genuine Sirt1-targeting therapeutic.

*Acteoside*, a prominent phenylethanoid glycoside derived from *Rehmanniae Radix*, exhibits a versatile pharmacological profile, encompassing anti-inflammatory, antioxidant, neuroprotective, and metabolic regulatory functions. These effects are largely underpinned by its capacity to modulate pivotal signaling cascades, including NF-κB, Nrf2, and MAPK pathways (Lim et al., 2021). In a compelling demonstration of its nephroprotective potential, Zhang et al. showed that *Acteoside* significantly lowered blood glucose, improved serum creatinine and BUN levels, and reduced 24-h total urinary protein in a rat model of diabetic nephropathy (unilateral nephrectomy combined with streptozotocin). Mechanistically, this was attributed to the inhibition of pyroptosis, evidenced by the downregulation of NLRP3, Caspase-1, IL-1β, and IL-18 via the PI3K/AKT/NF-κB pathway, ultimately mitigating podocyte injury and ameliorating renal pathology (Zhang SJ. et al., 2024). *Acteoside’s* anti-fibrotic effects on the kidney are shown in Figure 1.

In an adenine-induced nephropathy model, Acteoside was found to significantly reduce fibrotic lesions and inflammatory markers. Mechanistically, its effect was distinguished from other compounds. While *Acteoside* also converges on the Nrf2/NF-κB signaling axis, its mechanism is distinguished by the engagement of a novel upstream regulator. Seminal work by Wang et al. identified the aryl hydrocarbon receptor (AHR) as the apical mediator of *Acteoside*’s effects in both adenine-induced nephropathy and cellular models of fibrosis (Wang et al., 2025). Their initial observations revealed that *Acteoside* administration not only modulated the Nrf2 and NF-κB pathways but also concurrently suppressed AHR nuclear translocation and the expression of its canonical targets (CYP1A1, CYP1A2).

The causal link between AHR inhibition and the downstream immunomodulatory effects was elegantly established using pharmacological tools. Pre-treatment with an AHR inhibitor, CH223191, effectively abrogated *Acteoside*’s therapeutic efficacy. This molecular blockade prevented the suppression of NF-κB and the activation of the Nrf2 antioxidant program, thereby neutralizing the compound’s anti-fibrotic actions on markers like α-SMA and E-cadherin. Conversely, NF-κB inhibition did not alter AHR expression, confirming AHR’s position at the apex of this cascade. These data provide compelling evidence that *Acteoside* functions primarily as an AHR antagonist, which subsequently orchestrates the reciprocal regulation of the Nrf2 and NF-κB pathways. This discovery is profound, as it raises a critical and clinically relevant question: what is the endogenous AHR ligand driving renal fibrosis that *Acteoside* displaces or antagonizes? Identifying this ligand could unveil a novel pathogenic axis in CKD and solidify AHR as a high-value therapeutic target.

The recent study by Mao et al. (2023) provides the first pharmacological proof-of-concept for targeting this interface. They demonstrated that in obstructive nephropathy, acteoside treatment concurrently reduced macrophage infiltration (F4/80+) while systemically downregulating the entire HMGN1/TLR4/TREM-1 signaling cascade. This disruption of the damage-sensing machinery was directly linked to reduced pro-inflammatory cytokine production and attenuated fibrosis. This work is profound as it positions *Acteoside* as a dual-action agent, but it leaves a pivotal question of cellular specificity unresolved. Is *Acteoside* primarily cytoprotective, acting on tubular epithelial cells to limit the initial DAMP release and thus quell the inflammatory trigger at its source? Or is it predominantly immunomodulatory, acting directly on macrophages to render them hyporesponsive to HMGN1 stimulation by downregulating their TLR4/TREM-1 sensor apparatus? Understanding this cellular-level mechanism is crucial for defining *Acteoside* as either a tissue-stabilizing or a direct anti-inflammatory agent.

### 4.3 Alleviation of oxidative stress via the Nrf2 system

The pivotal role of the Nrf2/NF-κB signaling nexus in aristolochic acid I (AA-I)-induced nephrotoxicity was rigorously investigated by Liu et al. (2024). In murine and cellular models of AA-I-induced nephrotoxicity, Catalpol was shown to markedly improve renal function and halt fibrotic progression. This synergistic action was attributed to a dual protective mechanism: Catalpol robustly activates the cytoprotective Nrf2 antioxidant pathway while concurrently suppressing the pro-inflammatory and pro-fibrotic NF-κB signaling cascade. This synergistic action was shown to effectively halt fibrotic progression. These findings position the Nrf2/NF-κB axis as a critical regulatory node in renal fibrosis and highlight a key question for future research: what is the precise molecular mechanism of the crosstalk—direct or indirect—through which Catalpol-mediated Nrf2 activation leads to the downstream inhibition of NF-κB?

### 4.4 Restoration of autophagy and cellular homeostasis

The research by Zhou et al. (2024) is significant because it identifies *Acteoside* as a pharmacological agent capable of ameliorating fibrosis in diabetic nephropathy models. This protective effect was mechanistically demonstrated to be a result of reactivating the autophagy-lysosome pathway via the upregulation of TFEB. *Acteoside* reactivates the autophagy-lysosome pathway, thereby ameliorating fibrosis. The activation of TFEB by *Acteoside* likely mitigates fibrosis through a dual mechanism: not only by clearing ECM-related proteins but also by suppressing the source of sterile inflammation. We hypothesize that by enhancing autophagic clearance of DAMPs (e.g., damaged mitochondria), *Acteoside* prevents the activation of pro-inflammatory pattern recognition receptors, such as the NLRP3 inflammasome, within resident renal cells and infiltrating immune cells. Verifying this link between *Acteoside* -induced autophagy and the attenuation of inflammasome activity represents a critical and exciting frontier in understanding its immunomodulatory effects.

### 4.5 Regulation of other key signaling pathways

#### 4.5.1 Rehmannioside A inhibits the RAAS/MAPK14/IL-17 axis

The pathogenic interplay between the renin-angiotensin system (RAS) and the IL-17 inflammatory axis is a critical driver of hypertensive nephropathy. A recent landmark study has now positioned *ReA* as a potent inhibitor of this immunopathological nexus (Liu et al., 2025). While *ReA*’s capacity to modulate core signaling pathways like NF-κB and Nrf2 was previously established in non-renal models (Fu et al., 2022; Wang et al., 2022; Xiao et al., 2021), this work provides the first direct link between *ReA* and the immunopathology of renal disease. *ReA′s* anti-fibrotic effects on the kidney are shown in Figure 1.

The study revealed a multi-pronged therapeutic action. Systemically, *ReA* re-establishes RAS homeostasis. Locally, within the kidney, it quells the inflammatory and fibrotic response. The molecular linchpin of this effect was identified as MAPK14 (p38 MAPK). In a novel mechanism, *ReA* was shown to directly bind MAPK14, tagging it for ubiquitination and proteasomal degradation. This targeted protein degradation effectively short-circuits the Ang II-driven inflammatory cascade, preventing NF-κB activation and critically suppressing the production of IL-17—a key cytokine implicated in autoimmune and fibrotic kidney diseases.

The targeted degradation of MAPK14 in renal parenchymal cells is a profound discovery, but it raises a pivotal immunological question regarding cellular specificity. The study observed a reduction of IL-17 in kidney tissue, a cytokine primarily produced by Th17 cells. Therefore, does *ReA*’s effect stem solely from its action on renal cells, reducing the local inflammatory cues that attract and activate Th17 cells? Or, more intriguingly, does *ReA* also exert a direct, cell-intrinsic effect on infiltrating T cells, inhibiting their MAPK14-dependent differentiation into a pathogenic Th17 phenotype? Dissecting this interplay between parenchymal and immune cell responses is crucial for defining *ReA* as either a tissue-protective or a truly immunomodulatory agent.

#### 4.5.2 GABA modulates the local tubular GABAergic system

The inhibitory neurotransmitter *Gamma-Aminobutyric Acid* (*GABA*) reveals a non-canonical Reno protective mechanism. Its potent anti-fibrotic effect in a renal mass reduction model is mediated not systemically, but through the restoration of local, tubular *GABA* receptor expression (Sasaki et al., 2007). This finding points to the existence of an intrinsic, organ-specific signaling system that can be pharmacologically harnessed for tissue protection.

#### 4.5.3 Salidroside co-regulates Wnt, TLR4, and Sirt1/PGC-1α pathways

*Salidroside* epitomizes a multi-pronged therapeutic strategy. Its anti-fibrotic action is not confined to a single pathway but is executed by simultaneously dismantling at least three distinct pathogenic pillars: it suppresses aberrant developmental signaling (Wnt/β-catenin), quells innate immune activation (TLR4/NF-κB/MAPK), and reverses metabolic collapse by enhancing mitochondrial biogenesis (Sirt1/PGC-1α) (Xue et al., 2019; Shati and Alfaifi, 2020; Li et al., 2019).

Other compounds’ anti-fibrotic effects on the kidney are shown in Figure 1.

## 5 Conclusion and perspectives

The traditional remedy *Rehmanniae Radix* is not merely a source of natural products but a masterclass in immunomodulatory polypharmacology, deploying a multi-pronged strategy to dismantle the complex pathogenic network of renal fibrosis. This review has delineated how its primary natural products execute this strategy at multiple levels: they suppress canonical pro-fibrotic signaling (TGF-β/Smad), neutralize sterile inflammation by targeting innate immune sensors (AHR, TLR4/TREM-1), restore cellular homeostasis and quality control (TFEB-mediated autophagy, Nrf2), reverse metabolic collapse (Sirt1/PGC-1α), and rebalance systemic drivers of injury like the renin-angiotensin system. The concerted action of *Catalpol, Acteoside, ReA*, and other natural products provide a compelling molecular rationale for the herb’s historical efficacy and positions it as a rich resource for novel anti-fibrotic drug discovery.

### 5.1 Limitations of current research

Despite the promising data presented, this body of research, as a whole, possesses significant limitations that must be critically acknowledged. First and foremost, the evidence for the anti-fibrotic efficacy of these specific natural products is almost exclusively derived from preclinical (*in vivo* animal and *in vitro* cellular) models. Rigorous, well-controlled human clinical trials are conspicuously absent. The reviewed studies employ a wide array of animal models (e.g., UUO, adenine-induced, STZ-induced) and experimental conditions (e.g., varying doses, durations, and cell types), making direct comparison of the natural products’ potency and efficacy challenging. Superficial Pharmacological Data: As noted by reviewers, many studies provide insufficient detail on key pharmacological parameters, such as the tested dose ranges, positive/negative controls, or the specific experimental readouts used to validate claims. Finally, current research has focused almost entirely on single-agent analysis. The core concept of TCM polypharmacology—that these natural products may act synergistically—remains a hypothesis. It is unknown whether these natural products exhibit additive, synergistic, or even antagonistic interactions.

### 5.2 Future perspectives and unresolved questions

However, in illuminating these mechanisms, this body of work simultaneously brings a series of critical, high-resolution questions into sharp focus. These questions must now guide the next phase of research, moving beyond foundational discoveries toward translational application.

First, the challenge of cellular specificity must be addressed. We have seen that *Rehmannioside A* suppresses the production of IL-17, a cytokine primarily derived from Th17 cells. Does this reflect a direct, immunomodulatory effect on T-cell differentiation, or is it an indirect consequence of improving the local renal microenvironment? Similarly, does *Acteoside*’s inhibition of the HMGN1/TLR4/TREM-1 axis stem from a cytoprotective effect on tubular cells—preventing DAMP release at its source—or from a direct suppressive action on the myeloid cells that sense these alarmins? Dissecting this interplay between parenchymal and immune cell responses is a crucial frontier for understanding these natural products as either tissue-stabilizing or truly immunomodulatory agents.

Second, key upstream molecular mechanisms remain unresolved. How, precisely, does *Catalpol* activate Sirt1—is it a direct allosteric activator or an indirect modulator of the cellular NAD^+^ pool? What is the specific E3 ubiquitin ligase that *Rehmannioside A* recruits to mediate the targeted degradation of MAPK14? And, perhaps most profoundly, if *Acteoside* functions as an AHR antagonist to suppress fibrosis, what is the endogenous, pro-fibrotic ligand it displaces? Identifying this ligand could unveil an entirely new pathogenic axis in chronic kidney disease.

Finally, the paradigm must shift from single-agent analysis to understanding poly pharmacological synergy. The true therapeutic power of *Rehmanniae Radix* likely lies not in a single “magic bullet” but in the concerted action of its chemical portfolio. The critical next step is to investigate these combinatorial interactions. Does the metabolic reprogramming induced by salidroside, for instance, potentiate the anti-inflammatory efficacy of *Acteoside*? Answering such questions through network pharmacology and factorial experimental designs is essential to deconstruct the herb’s natural blueprint.

Ultimately, the path forward involves a journey from deconstruction to reconstruction: deconstructing this ancient remedy to understand its precise molecular and cellular logic, and then using that knowledge to reconstruct its wisdom into rationally designed, synergistic combination therapies. This approach will be pivotal in translating the promise of *Rehmanniae Radix* into precision-engineered therapeutics for the modern immunomorphology clinic.

## Abbreviations

AHR, Aryl Hydrocarbon Receptor; Ang II, Angiotensin II; AT1R, Angiotensin II Type 1 Receptor; CKD, Chronic Kidney Disease; DAMPs, Damage-Associated Molecular Patterns; ECM, Extracellular Matrix; GABA, Gamma-Aminobutyric Acid; GABA-R, GABA Receptor; HO-1, Heme Oxygenase-1; HMGN1, High-Mobility Group Nucleosome-binding protein 1; IFN-γ, Interferon-gamma; IL, Interleukin; LRP, Lipoprotein receptor-related protein; MAPK14, Mitogen-Activated Protein Kinase 14 (p38 MAPK); NF-κB, Nuclear Factor kappa-light-chain-enhancer of activated B cells; NQO1, NAD(P)H Quinone Dehydrogenase 1; Nrf2, Nuclear factor erythroid 2-related factor 2; p-Smad2/3, phosphorylated Smad2/3; PGC-1α, Peroxisome proliferator-activated receptor-gamma coactivator 1-alpha; RAAS, Renin-Angiotensin-Aldosterone System; ROS, Reactive Oxygen Species; Sirt1, Sirtuin 1; Smad, Small mothers against decapentaplegic; TEC, Tubular Epithelial Cell; TFEB, Transcription Factor EB; TGF-β, Transforming Growth Factor-beta; TGF-β-R, TGF-β Receptor; TLR4, Toll-like Receptor 4; TNF-α, Tumor Necrosis Factor-alpha; TREM-1, Triggering Receptor Expressed on Myeloid cells 1; Wnt, Wingless-related integration site.
